# “Eugenics is an integral aspect of our global scientific and political culture:” interview with Marius Turda

**DOI:** 10.1590/S0104-59702025000100050

**Published:** 2025-11-03

**Authors:** Marius Turda, Vivian Mannheimer

**Affiliations:** i Professor and director, Centre for Medical Humanities/School of Education, Humanities and Languages/Oxford Brookes University. Oxford – UK mturda@brookes.ac.uk; ii Journalist and postdoctoral researcher, Graduate Program in Communication/Pontifícia Universidade Católica do Rio de Janeiro. Rio de Janeiro – RJ – Brazil vmannheimer@gmail.com

**Keywords:** Eugenics, Racism, History, Twenty-first century, Anti-eugenics, Eugenia, Racismo, História, Século XXI, Antieugenia

## Abstract

Despite public condemnation after the defeat of Nazism, eugenic ideas continue to persist into the twenty-first century in different countries and contexts and with varying forms of intensity and support, as seen in events such as the covid-19 pandemic and the George Floyd case, discourse by contemporary far-right populist governments, and the 2024 US presidential election. This interview with Professor Marius Turda, a prominent scholar in the field of race, racism, and eugenics, presents his work and his itinerant exhibition entitled “We are not alone: legacies of eugenics,” aimed at specialised and non-academic audiences alike, and offers a historically informed account of our eugenic past, present, and future.

## Resumo

Apesar da condenação pública após a derrota do nazismo, ideias eugênicas persistem no século XXI, em diferentes países, contextos e com distintos graus de intensidade e apoio. Essa persistência pode ser observada em acontecimentos como a pandemia de covid-19, o assassinato de George Floyd, discursos de governos populistas de extrema-direita e a eleição presidencial dos Estados Unidos em 2024. Nesta entrevista, o professor Marius Turda – um dos principais estudiosos das questões de raça, racismo e eugenia – apresenta seu trabalho e a exposição itinerante “Não estamos sozinhos: os legados da eugenia”, voltada ao público acadêmico e à comunidade, propondo uma reflexão historicamente fundamentada sobre esses legados no passado, no presente e para o futuro.

Eugenics is far from being a thing of the past. The search for “improvement of the race,” which had its most horrendous expression in the Holocaust, survived the defeat of Nazism and left legacies throughout the twentieth century and up to the present day, as explained in this interview with Professor Marius Turda, director of the Centre for Medical Humanities at the School of Education, Humanities, and Languages at Oxford Brookes University.

Turda has authored, co-authored, and edited more than 25 books on eugenics, race, and racism. They include *Modernism and eugenics*, *Latin eugenics in comparative perspective*, *The history of East-Central European eugenics, 1900-1945: sources and commentaries*, and *A cultural history of race*, published in six volumes by Bloomsbury Academic in 2021. His latest book is *In search of the perfect Romanian: national specificity, racial degeneration and social selection in Modern Romania*, published in 2024 (English edition forthcoming in 2026).

According to Turda, eugenic ideas have persisted into the twenty-first century in different shapes and forms, with state and professional support. From the institutionalized version that existed until the mid-1970s to various sterilization programs in the United States, Europe and elsewhere, the legacies of eugenics based on the old argument about “lives unworthy of living” or “less worthy” could also be detected during the covid-19 pandemic, in systemic racialization of people of colour (as tragically exemplified by the murder of George Floyd), and in the current debates on medically assisted suicide in North America, Britain, and Europe.

Additionally, Marius Turda is the curator of the itinerant exhibition “We are not alone: legacies of eugenics,” which he brought to Fiocruz [Fundação Oswaldo Cruz] in April 2024.^1^ The exhibition explores eugenic ideas in different national and international contexts, contributes to the resurgence of an anti-racist movement, and affirms the fundamental right to human difference and the need to combat eugenics in all its forms.

*How did you start your research on eugenics?*

I began working on the history of eugenics in 2002 after I completed my PhD. At the time, I was interested in the history of eugenics in Hungary and, more broadly, in East-Central Europe. It took a long time to complete the research, however, as many of the archives were unknown and the secondary literature was sparse. My aim was to demonstrate – and I hope I did it convincingly in the books that I’ve published on the history of eugenics in this region – that there were emerging eugenic movements in the region by the early 1900s and that these flourished during the interwar period, in some cases with tragic consequences, including the Holocaust. It is clear that the trajectory of eugenics in East-Central Europe during the first half of the twentieth century mirrored trajectories elsewhere, particularly in Germany, Britain, United States, France and Italy, but it is also clear that eugenics had emerged as an attempt to provide answers to a variety of local problems conditioned by the specific social, economic, religious and cultural particularities of this region as well as political developments, most notably the creation of nation-states after 1918-1919.

The history of eugenics, racism and the history of nation-state building in East-Central Europe are closely interlinked. We need to see the fascination with eugenics moulding not only strategies of public health and social hygiene but also nationalist strategies of ethnic belonging and exclusion. For both eugenics and nationalism, a clearly motivating force was that of the identification and belonging of individuals, communities, nations, races, and so on. Old and new problems facing East-Central Europe after First World War were reconfigured in terms of the eugenic health of the nation, and the eugenicists were uncompromising supporters of this process of redefinition of the national community alongside biological criteria.

My research pointed out that eugenics provided professionals and state officials in East-Central European countries with a strong scientific foundation on which to erect programmes of social and biological improvement. By suggesting that the development of modern nation-states in this region be read through the lens of eugenics, I hope to have challenged the putative peripherality of this region, and the neglect it had experienced for decades, not only from scholars of eugenics, but also from scholars of nationalism, racism and antisemitism.

*Your book Latin eugenics in comparative perspective was published 11 years ago. Since then, what have been the new discussions, topics, or ideas on Latin eugenics, and what are the main differences between it and eugenics in other parts of the world?*

Indeed, it’s been 11 years since the publication of *Latin eugenics in comparative perspective*, a book which was meant to complement William H. Schneider’s *Quality and quantity: the quest for biological regeneration in twentieth century France* (published in 1990) and Nancy L. Stepan’s influential *The hour of eugenics: race, gender, and nation in Latin America* (published in 1991). Both authors provided a much-needed revision of conventional interpretations of eugenics that focused predominantly on Anglo-Atlantic and German experiences. In this book, I wanted to show that eugenics was not only a self-styled scientific interpretation of human improvement based on various theories of heredity, but also a worldview which absorbed its sustenance from cultural traditions, linguistic affiliations and religious norms. A number of countries in Europe and South America such as France, Italy, Spain, Portugal, Romania, Argentina, Brazil, Chile and Uruguay committed to a universal “Latin” culture, based on the notion of a common historical and intellectual heritage. Religious, cultural and linguistic commonalities were seen as a source for political inspiration and cultural renewal, but also as a model for racial harmony and assimilation. The realm of a shared “Latin” culture provided a platform upon which eugenicists, demographers, social hygienists and child welfare activists in these countries built their theories of a distinctive form of eugenics, serving the cultural and historical particularities of their own societies. For them, a “Latin” version of eugenics appeared to offer a progressive programme of social and medical reform, alongside pronatalist campaigns to educate the population about the importance of large and healthy families.

**Figure 1:**
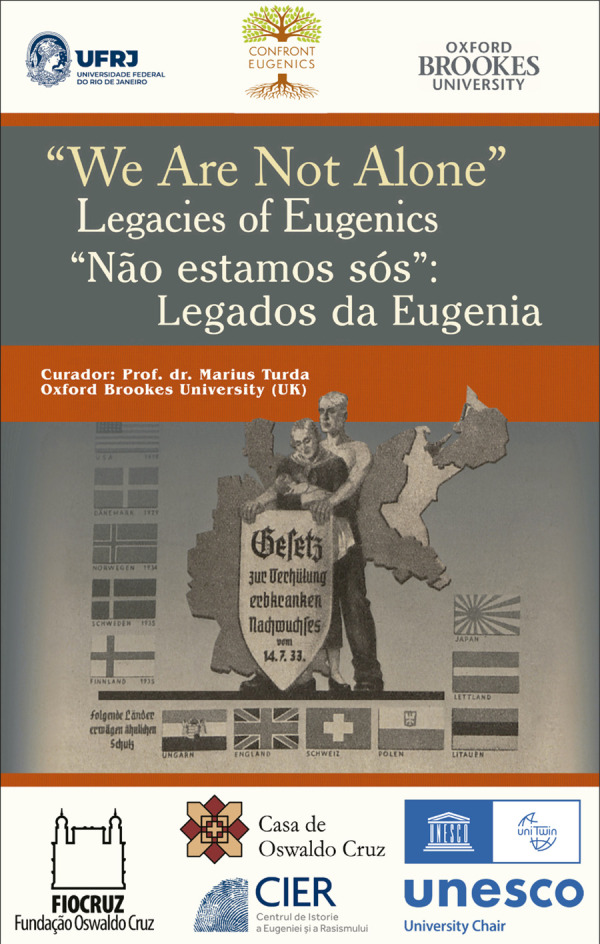
: Exhibition poster for "We are not alone: legacies of eugenics", at Fiocruz in April 2024 (Source: Artwork made by Marius Turda)

In the 1930s, a “Latin” ecumenism was also promoted by these eugenicists who felt that the so-called Nordic or Anglo-Saxon model of eugenics promoted in Germany, the United States, Britain and the Scandinavian countries was too invasive, too coercive, and focused too much on the prevention of certain individuals from reproduction with the presumed aim of improving and strengthening the “race.” There was also consensus amongst “Latin” eugenicists as to their criticism of compulsory sterilisation, birth control, abortion and (last but not least) racism, particularly its Nazi variant emerging after 1933.

In highlighting these aspects of eugenics I was, I think, in agreement with scholars working on the history of eugenics in South America such as Marisa Miranda, Gustavo Vallejo, Yolanda Eraso, Andrés H. Reggiani, Robert Wegner, and Vanderlei Sebastião de Souza, who had already engaged with these topics in various articles. New sources have since become available that enable historians to explore different aspects of “Latin” eugenics. There are some fine recent studies (by authors such as Ramón Castejón Bolea, Chiara Beccalossi, Nora E. Jaffary, François Secco, Edivaldo G. Junior, Sarah Walsh, Paola Dogliotti, Elizabeth Ortega, María José Beltrán, Myriam Mitjavila, Pietra Diwan, Luc Berlivet and Raúl Necochea López), which offer new ways of understanding the lasting legacy of eugenics on these countries’ demographic and family policies, preventive medicine, social hygiene and public health.

*What happened to eugenics after the Second World War? How can we relate it to contemporary events such as the George Floyd case, the covid-19 pandemic, and far-right populist governments?*

Undoubtedly, the association with the Nazi racial state dealt a severe blow to state-enforced eugenic programmes of sterilisation and ethnic cleansing. But eugenics as a social and medical movement survived the defeat of Nazism. In the United States, Britain, Argentina, Brazil and elsewhere, scientists continued to endorse eugenics during the 1950s and 1960s in relation to issues such as contraception, family planning and voluntary sterilisation, all affecting people who were primarily non-white, poor, or working class. Eugenics may have been compromised in the eyes of politicians, but not in the eyes of ordinary people. As the British psychiatrist C.P. Blacker, General Secretary of the Eugenics Society, argued in a book published in 1952, eugenics is no longer a monopoly of experts; it enters the life of the common man whose aspirations and beliefs determine how far eugenic principles can at any time affect social practice (Blacker, 1952). The institutionalisation of eugenics also continued beyond the Second World War. For instance, the Argentine Eugenics Society was established in 1945, and the Greek Eugenics Society was formed only in 1953. The American Eugenics Society was dissolved officially in 1972, and it was not until 1989 that the Eugenics Society in Britain changed its name to The Galton Institute.

During the 1960s, crude notions of an inherited “germ plasm” were replaced by the more specific “genes.” And again, the scientific identification of “good” and “bad” genes and their social validation became intertwined. The architects of post-Second World War welfare, health care and social assistance programmes lamented the difficulty of fixing the outcome of successive generations of unfortunate mating choices through education and environmental improvement. Instead, they continued to look to eugenic solutions such as institutionalisation and sterilisation.

Most troublingly, until the mid-1970s, sterilisation programmes in the United States robbed significant numbers of Black, Indigenous, and Hispanic women of their ability to have children. It ruined women’s reproductive health. These women continue to be affected by similar eugenic practices and their legacies in the present day. Just as disturbingly, the United States saw recent revelations of the involuntary hysterectomy of migrants in US immigration custody, instances which echo the sterilisation of Roma women in Czechoslovakia during communism and beyond.

The testimonies of victims of sterilisation worldwide ensure that eugenics can never be relegated to the past. It demands that we confront the legacies of eugenics that are in many ways still with us today. For instance, in Denmark, state-sponsored genetic testing for “Down syndrome” was combined with the omnipresent stigma associated with this genetic disorder. When introduced in the late 1970s, the eugenic undertones of this method were clearly stated: to prevent the birth of someone with disability was cheaper than to institutionalise and care for them. Since then, non-invasive prenatal testing, preimplantation genetic diagnosis and newborn sequencing have become widely accessible, but anxieties about their eugenic implications have not disappeared. Many prospective parents continue to worry that it is only possible for children to be born and live without significant stigma if they are considered “normal” or born to individuals and in families the state and society consider “normal.” These children are “normal” only if they conform to the eugenic ideal of a healthy individual, free of “disease” and “disorder,” subscribing to a narrow standard of beauty, ability and worth which are impossible to achieve, financially bankrupt to implement, morally problematic, and ultimately based upon aesthetic and cultural standards which themselves were produced by Western scientific racism.

Though differing in forms, intensity and levels of state and professional support, eugenic ideas and practices have persisted into the twenty-first century. The legacies of eugenics can also be detected in the ways in which the covid-19 pandemic has reinforced forms of discrimination based on one’s health, age, gender, and ethnic origin. The old eugenic argument about “lives unworthy of living” or “less worthy” have returned to the public mind, as governments were struggling to draw the line between prevention and protection. Another recent example is the use of eugenic typologies and offensive eugenic language to describe political opponents, as it happened during the last presidential election in the United States.

The rise of biological determinism in our society and politics cannot be ignored. Racial prejudice, biological labelling, and chauvinistic descriptions of ethnic, social, and sexual minorities are as effective now as they were in the past. Consider, for instance, politicians in the United States, Hungary, Romania, Italy, Britain and Poland who speak of immigrants and desperate refugees arriving at their countries’ borders as representing “a radically different culture” and that to grant them entrance means to open the gates of ethnic miscegenation and racial replacement. Racialised and eugenic versions of Europeanness and whiteness are to be used in the public sphere alongside an aggressive rhetoric of national protectionism and ethnicity. National communities continue to be perceived by far-right populist governments across the world as organic, homogenous entities in need of eugenic protection from “undesired” individuals. The spectre of eugenics therefore continues to loom large not just in genomics and CRISPR gene editing but also in our political discourse and practice.

*What are you working on, and what are your research interests?*

Last year I published a book on national character, eugenics and biopolitics in modern Romania entitled *In search of the perfect Romanian*, and now I am completing a book on scientific racism in Hungary between 1880s and 1940s. In 2026, I hope to continue with my work and exhibition on the global legacies of eugenics and then, hopefully, write a book on the process of eugenic dehumanisation. With its objectifying and stigmatising gaze, eugenics had distinctly shaped the modern ideal of physical fitness and intellectual superiority and with it of an able society, one in which those individuals who were seen as embodying a different humanity were relegated to institutions, special educational programmes and marginal social spaces. These individuals were not considered valuable members of the national body politic. Dehumanisation enabled strategic definitions of the scope and substance of eugenics on the basis of individually and collectively articulated needs. I want to write about the eugenic practices attached to the naming of individuals considered to be “different” but also about health strategies, systems of treatment and care which were introduced in the past century to produce a new conceptualisation of disability that is still with us today.

*Can you talk about the exhibition “We are not alone: legacies of eugenics” you brought to Fiocruz?*

In September 2021, I curated an exhibition on the history and legacies of eugenics at the Wiener Holocaust Library to mark a century since the Second International Eugenics Congress was organised at the American Museum of Natural History in New York. Since then, the exhibition has travelled to Romania, Poland, Sweden, United States, Switzerland, Serbia, Finland and Brazil, at Fiocruz and Casa Preta da Maré in Rio de Janeiro. In Britain the exhibition has been displayed in Sheffield, Bristol, London – at the Royal College of Psychiatrists and at the Institute of Education of the University College London, and at the Museum of Oxford. A digital catalogue is now available at https://www.brookes.ac.uk/research/units/hss/centres/centre-for-medical-humanities#flipbook.

The first aim of the exhibition is to reveal the shifting and fluid meanings that characterised eugenic ideas of human betterment in different national and international contexts and to offer a historically informed account of our eugenic past, present, and future, balancing various elements of continuity and discontinuity, of idiosyncrasy and similarity. The second aim is perhaps more important. I want to say to the visitors that without continued education about and engagement with the history eugenics, as well as its public condemnation, we are not effectively challenging dominant narratives about the national past, nor are we effectively attending to deep-seated racism, hostility towards immigrants and foreigners, and discrimination against people of colour and those with learning disabilities. As I show in this exhibition, eugenics is an integral aspect of our global scientific and political culture, one in which the state and the society embarked on an unprecedented quest to create an idealised individual offered by the promises of modern science and medicine.

On a personal note, the exhibition has radically changed my approach to the history of eugenics. To curate it meant going through very diverse archival materials… but this was the easy part! It was more difficult to adapt to a very different way of thinking about the current relevance of my academic work. As a scholar of eugenics and racism, I wanted to contribute to the resurgence of an anti-racist movement of reckoning with the past, after the killing of George Floyd, in 2020. What is now needed is to place eugenics within a new framework, and see it as interconnected with other paradigms within global racism, social and health inequities. We must put an end to the eugenic dehumanisation of many people of colour, of those individuals with learning disabilities, and so on. We must stop the eugenic rationalisation of their supposed “backwardness,” “inferiority,” and of their “flawed” identity.

Finally, what is also needed is for scholars to come out and express a personal commitment, their own investment in the study of eugenics and in combatting its legacies in today’s world. Therefore, to combat eugenics and scientific racism there must be an even broader, bolder large-scale effort to rehumanise individuals and groups in science and beyond. An anti-eugenics movement should include the compensation of victims of sterilisation as well as educational strategies and the empowerment of indigenous and minority communities.

During my visit at UFRJ [Universidade Federal do Rio de Janeiro] and Fiocruz in April 2024, I learned from Brazilian students and scholars, and I hope I gave something in return for their hospitality and generous collegiality: an exhibition which affirms the fundamental right to human difference and the need to combat eugenics in all of its forms.

## NOTES

1 The exhibition that provided the opportunity for this interview was a collaboration with Brazilian researchers. Marius Turda visited Rio de Janeiro at the invitation of Gracyelle Costa Ferreira (Escola de Serviço Social/UFRJ), Juliana Manzoni (COC/Fiocruz), and Robert Wegner (COC/Fiocruz). The 12 panels that comprised the exhibition were translated to Portuguese by the team that works on the research project entitled “História da genética e de seus impactos: raça, racismo e população no Brasil” [“The history of genetics and its impacts: race, racism, and population in Brazil”] (Proep/CNPq/Fiocruz/COC) and the PPGHCS doctoral candidates Ana Carolina Correa Ferraz, Angelo Nicoladeli and Bernardo Felberg. The design and layout of the Portuguese-language version of the panels was done by the ASCOM/COC team, especially Silmara Mansur.
